# Recombinant Haplotypes Narrow the ARMS2/HTRA1 Association Signal for Age-Related Macular Degeneration

**DOI:** 10.1534/genetics.116.195966

**Published:** 2016-11-21

**Authors:** Felix Grassmann, Iris M. Heid, Bernhard H. F. Weber, International AMD Genomics Consortium (IAMDGC)

**Affiliations:** * Institute for Human Genetics, University of Regensburg, D-93053 Regensburg, Germany; † Department of Genetic Epidemiology, University of Regensburg, D-93053 Regensburg, Germany

**Keywords:** age-related macular degeneration, genetic association studies, linkage disequilibrium, haplotypes, *ARMS2*/*HTRA1* gene locus

## Abstract

Age-related macular degeneration (AMD) is the leading cause of blindness in ageing societies, triggered by both environmental and genetic factors. The strongest genetic signal for AMD with odds ratios of up to 2.8 per adverse allele was found previously over a chromosomal region in 10q26 harboring two genes, *ARMS2* and *HTRA1*, although with little knowledge as to which gene or genetic variation is functionally relevant to AMD pathology. In this study, we analyzed rare recombinant haplotypes in 16,144 AMD cases and 17,832 controls from the International AMD Genomics Consortium and identified variants in *ARMS2* but not *HTRA1* to exclusively carry the AMD risk with *P*-values between 1.0 × 10^−773^ and 6.7 × 10^−5^. This now allows prioritization of the gene of interest for subsequent functional studies.

AGE-RELATED macular degeneration (AMD) is a prevalent cause of severe vision loss in ageing societies with a strong component of genetic predisposition. Genome-wide association studies (GWASs) and large-scale resequencing initiatives have identified a large number of single nucleotide variants (SNVs) enriched in complement and complement-related genes that confer a strong risk for AMD. Recently, the International AMD Genomics Consortium (IAMDGC) (Fritsche *et al.* 2016) reported 34 independent AMD risk loci, together explaining ∼50% of disease heritability.

Among the strongest loci associated with AMD are the complement factor H (*CFH*) locus on chromosome 1q32 and a region on chromosome 10q26 harboring two genes, namely age-related maculopathy susceptibility 2 (*ARMS2*) and HtrA serine peptidase 1 (*HTRA1*) (Jakobsdottir *et al.* 2005; Rivera *et al.* 2005). While the causative genes at the *CFH* site appear undisputed, the *ARMS2/HTRA1* region proved notoriously difficult to dissect by statistical means as the chromosomal region displays correlated variants in high linkage disequilibrium (LD) (Dewan *et al.* 2006; Fritsche *et al.* 2008; Kanda *et al.* 2010; Yang *et al.* 2010; Friedrich *et al.* 2011, 2015). In addition, as both *ARMS2* and *HTRA1* harbor functional variants that can be related to relevant disease processes, so far it is unclear to which gene the observed disease association can functionally be attributed (Dewan *et al.* 2006; Fritsche *et al.* 2008; Cheng *et al.* 2013; Friedrich *et al.* 2015).

Despite the strong LD in the *ARMS2/HTRA1* interval, the region still exhibits some level of recombination, resulting in rare recombinant haplotypes. Similar to gene mapping in monogenic diseases, recombinant haplotypes can be helpful in dissecting a disease-associated genomic region. To this end, we used the currently largest data set on AMD genetics including >33,000 genotyped individuals (Fritsche *et al.* 2016) and analyzed the rare, but informative recombinant haplotypes on 10q26 to define a minimal set of variants associated with AMD.

## Materials and Methods

### Ethics statement

The study followed the tenets of the Declaration of Helsinki and was approved by the local ethics review board at participating sites, as previously described (Fritsche *et al.* 2016). Informed written consent was obtained from each patient after explanation of the nature and possible consequences of the study.

### Study data and data availability

The genotypes of this study are available from the Database of Genotypes and Phenotypes (dbGAP) under accession phs001039.v1.p1 while GWAS summary statistics are available at http://amdgenetics.org/. Our data consist of 16,144 late-stage AMD cases and 17,832 AMD-free controls from European ancestry, all unrelated, as published previously (Fritsche *et al.* 2016). Inclusion and exclusion criteria as well as detailed information on ophthalmological grading and quality control of genetic data and imputation are given elsewhere (Fritsche *et al.* 2016). Genotyping and imputation using the 1000 Genomes (1000G) reference panel (Abecasis *et al.* 2012) was performed as described previously (Fritsche *et al.* 2016). Briefly, we extracted the genotypes of all variants in the *ARMS2/HTRA* locus, defined as the region of 1.25 million base pairs (bp) around the previously described lead variant rs3750846. The extracted genotypes were phased with ShapeIt2 following standard settings (O’Connell *et al.* 2014). The resulting haplotypes were then used for imputation with IMPUTE2 (Rahpeymai *et al.* 2006), utilizing the 1000G reference panel. Upon imputation, IMPUTE2 generates best-guess imputed haplotypes, which can be outputted using the -haps command. In addition, the imputed genotypes were coded as dosage data ranging from 0 to 2 for single variant association testing as well as mediation analyses. In total, 3,446 variants were either genotyped or could be imputed reliably in the *ARMS2/HTRA1* region.

Several DNA specimens had been genotyped after whole genome amplification (WGA). To account for possible confounding effects of WGA, a categorical variable “WGA” was computed, indicating the presence or absence of WGA in all samples. Additionally, the first two principle components (PC1 and PC2) were computed from all genotyped variants as described previously (Fritsche *et al.* 2016). All logistic regression models were adjusted for WGA and PC1 and PC2 to account for different DNA source and potential population stratification.

### Searching for additional signals at the *ARMS2/HTRA1* locus

All statistical procedures were carried out as implemented in R. Step-wise conditional logistic regression analysis on the imputed dosages was used previously and failed to detect additional independent signals at the *ARMS2/HTRA1* locus (Fritsche *et al.* 2016). We also aimed to exclude the possibility that the main signal represented by rs3750846 was explained by two causal variants where the risk-carrying alleles are inherited together with one of the rs3750846 alleles. In such a scenario, the two causal variants would represent two or more haplotypes, which would be tagged by one of the alleles of rs3750846 (Grassmann *et al.* 2012; Fritsche *et al.* 2016). We used mediation analysis to test each pair of variants in the region for jointly explaining the main signal: the β-estimate of the main variant (the rs3750846) in a logistic regression model without adjusting for the pair is compared to the respective β-estimate with adjusting for the pair of variants (Imai *et al.* 2010), using the *mediate* function from the *mediation* package in R (Tingley *et al.* 2014).

For all analyses, we utilized the genotypes determined experimentally or the dosages in the case of imputed variants. In the case of a significant mediation, the effect size of the main variant would be expected to drop strongly and significantly. To obtain reliable nonparametric *P*-value estimates, we calculated 1000 bootstrap replicates and adjusted the resulting *P*-values according to the false discovery rate. To reduce the complexity of the mediation analysis, we first extracted variants in linkage with rs3750846 by computing *D*′ using the haplotypes obtained from the imputed genotypes and extracted all variants with *D*′ > 0.8. This should effectively capture the relevant common haplotypes tagged by rs3750846.

### Best-guess haplotype association analyses

To further investigate the AMD-associated haplotypes at the *ARMS2/HTRA1* locus, we extracted the relevant variants. To capture the most likely causal variant (van de Bunt *et al.* 2015), we included variants that were correlated to rs3750846 (*R*^2^ > 0.8) as well as all variants that were included in the 99% credible set of associated variants (Maller *et al.* 2012; Fritsche *et al.* 2016). The 99% credible set of associated variants was computed from the *Z*-scores of all variants at this locus (Kichaev *et al.* 2014), effectively capturing the variants with the strongest evidence for association. The best-guess haplotypes defined by these variants (*i.e.*, haplotypes that carry alleles of these variants) were investigated for their association with AMD using multivariable logistic regression models including all haplotypes with reasonable counts (≥34) in the study sample except the nonrisk allele-carrying haplotype (H0), which served as reference. We assessed the association of the haplotypes using a logistic regression model including all haplotypes. Haplotype H0, which carried exclusively nonrisk-increasing alleles served as baseline. We excluded variants to be disease associated if either the nonrisk-increasing alleles (protective alleles) of the variant were present on haplotypes that increase the risk for AMD or if the risk-increasing alleles were present on protective haplotypes or on haplotypes not associated with AMD.

### Accounting for phase uncertainty

To determine haplotype phase, the calculation of several plausible haplotypes for each individual is required. This process is repeated many times and eventually returns the best-guess haplotypes for each individual. It thus is possible that the estimated best-guess haplotypes are only slightly more likely than other haplotypes and that random effects may come into play. To account for this uncertainty, we repeated the phasing of the haplotypes and the subsequent imputation of the *ARMS2/HTRA1* locus 100 times and mapped the occurrence of the resulting haplotypes to the best-guess haplotypes. As such, each individual is characterized by the frequency or dosage of the 13 haplotypes (ranging from 0 to 2). The haplotype dosages were then analyzed for their association with AMD using the function haplo.glm from the package haplo.stats in R (Lake *et al.* 2003).

### Data availability

The authors state that all data necessary for confirming the conclusions presented in the article are represented fully within the article.

## Results

The present study included 16,144 late-stage AMD cases and 17,832 AMD-free controls with both groups of European ancestry (Fritsche *et al.* 2016). Variant and sample quality control as well as imputation based on the 1000G reference panel (Abecasis *et al.* 2012) was reported previously (Fritsche *et al.* 2016). Variant rs3750846 residing within intron 1 of *ARMS2* exhibited the strongest association with AMD on 10q26 and is referred to as lead variant (Fritsche *et al.* 2016). We defined the *ARMS2/HTRA1* locus as a region within 1.25 million base pairs around rs3750846 to assure the capture of all potentially correlated and associated variants.

Initially, we explored the number of causal variants explaining the association signal at *ARMS2/HTRA1*. When applying stepwise logistic regression as described previously (Fritsche *et al.* 2016), there was only one but no independent second signal at this locus. There was also no pair of variants that jointly explained the main signal when applying mediator analyses. Together, these findings suggested that the association at this locus was conferred to by a single haplotype tagged by the lead variant rs3750846.

**Figure 1:**
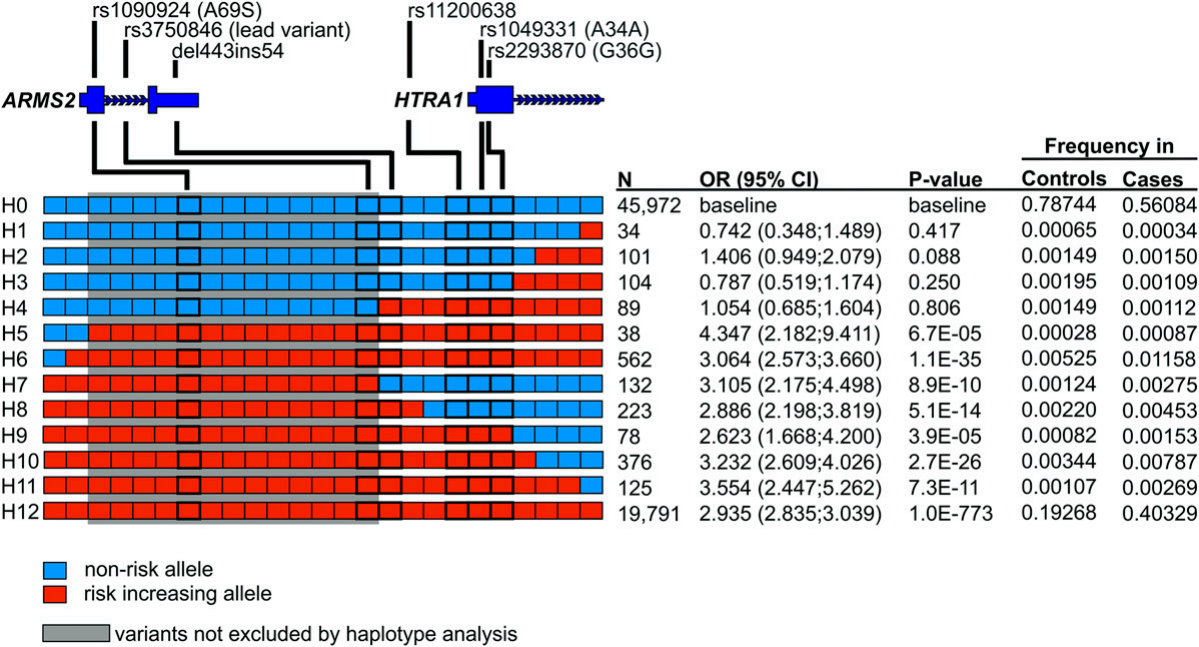
Delineating associated haplotypes at the *ARMS2/HTRA1* locus. Shown are 13 haplotypes defined by 25 variants that were either correlated with the lead variant rs3750846 (*R*^2^ > 0.8) or in the 99% credible set of associated variants (Fritsche *et al.* 2016) (see also Table 1). *P*-values are given based on a logistic regression model including H1–H12 as covariates and H0 carrying exclusively nonrisk alleles as reference. AMD risk-increasing alleles are colored in red and the protective alleles in blue. In the case in which a haplotype is carrying the risk-increasing allele of a variant but is not significantly associated with AMD, this variant is regarded not to be associated with disease risk. Similarly, if a haplotype carrying the protective variant allele is increasing the risk for the disease, we concluded that this variant is not associated with AMD risk. The minimal set of risk-associated variants (gray box) includes 13 variants not excluded by the haplotype analysis and located exclusively at the immediate *ARMS2* locus. Thin dark blue boxes represent the UTR; thick dark blue boxes represent the coding region of *ARMS2* as well as the first exon of *HTRA1*. Gene sizes and relative positions are not to scale. Intronic sequences are given by dark blue arrows indicating the direction of transcription. Each square as part of a haplotype represents a single variant, ordered by chromosomal position from centromere (left) to telomere (right) (see also Table 1). Also shown is the number of observed best-guess haplotypes (N), the odds ratio (OR), and 95% confidence intervals (95% C.I.) as well as the frequency of the best-guess haplotypes H0–H12 in cases and controls.

We then narrowed the region of interest by haplotype analysis focusing on highly correlated variants. The best-guess haplotypes for the locus were derived and their complexity was reduced by focusing on 25 variants that were (i) highly correlated with rs3750846 (*R*^2^ > 0.8) or (ii) in the 99% credible set of associated variants (Maller *et al.* 2012; van de Bunt *et al.* 2015; Fritsche *et al.* 2016) (Table 1). This resulted in 13 haplotypes that were counted at least 34 times in the study sample and thus resulted in a haplotype frequency ≥0.05% (Figure 1). Twelve haplotypes were included in a haplotype association analysis by logistic regression modeling with the common nonrisk allele representing haplotype H0 as reference (Figure 1). As expected, the common haplotype carrying all risk-increasing alleles (H12) was strongly associated with AMD (*P* < 10^−50^).

**Table 1:** Candidate variants at the *ARMS2/HTRA1* locus on 10q26 correlated with rs3750846 (*R*^2^ > 0.8) or part of the 99% credible set of associated variants

Position on Chr. 10 (bp)	rs ID	Nonrisk allele	Risk allele	Location/ consequence	−Log10 *P*-value	PPA*^a^*	*R*^2^*^b^*	*D*′*^b^*	OR (95% C.I.)*^c^*	Excluded
124,203,787	rs61871744	T	C	Intergenic	741.2	1.83×10^−42^	0.952	0.995	2.787 (2.693;2.885)	Yes
124,209,684	rs11200630	T	C	Intergenic	774.5	3.56×10^−9^	0.989	0.999	2.798 (2.706;2.894)	Yes
124,210,369	rs61871745	G	A	Intergenic	780.4	0.003	0.996	0.999	2.804 (2.711;2.900)	No
124,211,536	rs11200632	A	G	Intergenic	780.2	0.002	0.998	0.999	2.796 (2.703;2.891)	No
124,211,596	rs11200633	C	T	Intergenic	780.3	0.002	0.998	0.999	2.795 (2.703;2.890)	No
124,212,913	rs61871746	T	C	Intergenic	781.3	0.024	0.998	1.000	2.794 (2.702;2.889)	No
124,213,046	rs61871747	C	T	Intergenic	781.5	0.033	0.998	1.000	2.793 (2.701;2.888)	No
124,214,448*^d^*	rs10490924	G	T	ARMS2: p.A69S	781.8	0.076	0.998	1.000	2.786 (2.694;2.881)	No
124,214,600	10:124214600	G	GGT	ARMS2: intronic	781.9	0.091	0.998	1.000	2.787 (2.695;2.881)	No
124,214,976	rs36212731	G	T	ARMS2: intronic	781.9	0.094	0.999	1.000	2.787 (2.695;2.881)	No
124,215,198*^d^*	rs36212732	A	G	ARMS2: intronic	781.9	0.085	0.999	1.000	2.786 (2.695;2.881)	No
124,215,211	rs36212733	T	C	ARMS2: intronic	782.0	0.110	0.999	1.000	2.787 (2.695;2.881)	No
124,215,315	rs3750848	T	G	ARMS2: intronic	782.1	0.139	0.999	1.000	2.787 (2.695;2.882)	No
124,215,421*^d^*	rs3750847	C	T	ARMS2: intronic	782.2	0.168	0.999	1.000	2.787 (2.695;2.882)	No
124,215,565	rs3750846	T	C	ARMS2: intronic	782.2	0.173	1.000	1.000	2.787 (2.695;2.882)	No
124,216,820	esv2663177	443 bp	54 bp	ARMS2: UTR del442ins54	770.0	1.02×10^−13^	0.981	0.991	2.762 (2.671;2.856)	Yes
124,219,275*^d^*	rs3793917	C	G	Intergenic	768.0	1.10×10^−15^	0.979	0.990	2.756 (2.665;2.849)	Yes
124,220,061	rs3763764	A	G	Intergenic	759.6	4.05×10^−24^	0.965	0.989	2.746 (2.656;2.839)	Yes
124,220,544*^d^*	rs11200638	G	A	HTRA1: Promoter	755.9	9.29×10^−28^	0.963	0.988	2.737 (2.647;2.830)	Yes
124,221,270	rs1049331	C	T	HTRA1: p.A34A	755.6	5.06×10^−28^	0.961	0.987	2.744 (2.654;2.838)	Yes
124,221,276	rs2293870	G	T	HTRA1: p.G36G	755.5	3.44×10^−28^	0.961	0.987	2.744 (2.654;2.838)	Yes
124,226,630	rs2284665	G	T	HTRA1: intronic	742.4	3.10×10^−41^	0.933	0.974	2.724 (2.634;2.817)	Yes
124,230,024	rs58077526	A	C	HTRA1: intronic	716.7	5.71×10^−67^	0.896	0.958	2.672 (2.584;2.763)	Yes
124,231,464	rs932275	G	A	HTRA1: intronic	716.7	5.23×10^−67^	0.899	0.962	2.678 (2.590;2.770)	Yes
124,234,037	rs2142308	G	C	HTRA1: intronic	711.1	1.45E−72	0.888	0.956	2.666 (2.578;2.756)	Yes

a Posterior probability of association.

b Linkage to the top variant rs3750846.

c Odds ratio and 95% C.I. of risk increasing allele.

d Directly genotyped variant.

Two additional findings were of particular interest when focusing on the rare recombinant haplotypes: (1) haplotypes H1–H4 with risk-increasing alleles exclusively at the variants downstream of rs3750846 were not associated with AMD (*P* > 0.05) (Figure 1), while (2) haplotypes H7–H11 with the nonrisk alleles at the variants downstream of rs3750846 were highly significantly associated with AMD (*P* from <10^−50^ to 6.68 × 10^−5^). In addition, the first two variants upstream of *ARMS2* (rs61871744 and rs11200630) were also not associated with AMD, since haplotypes without the risk-increasing alleles at the two variants were associated with increased disease risk. Together, these findings reveal that variants downstream of the lead variant rs3750846 do not contribute to AMD risk and that the most likely candidates driving the AMD association are 13 variants in and immediately upstream of the *ARMS2* gene. A further narrowing of the refined AMD-associated interval would require an even larger data set due to the low rate of recombination between the local variants at and around *ARMS2*.

Our estimation of haplotypes and subsequent imputations relied on the calculation of best-guess haplotypes derived from several likely haplotypes calculated over several (internal) iterations. However, some of those recombinant haplotypes could in fact be better represented by less likely haplotypes, which were not chosen initially due to random effects. We therefore repeated the phasing and imputation 100 times to account for phasing uncertainty. The resulting haplotype occurrences were mapped to the 13 best-guess haplotypes and analyzed using logistic regression. The results of this analysis were similar to the results obtained from the best-guess haplotypes (Supplemental Material, Table S1), indicating that the reconstructed best-guess haplotypes are robust and likely represent the true haplotype structure in our cohort.

## Discussion

Since its initial reporting, the *ARMS2/HTRA1* region has been a point of controversy as to which gene is causally linked to AMD pathogenesis (Yang *et al.* 2006; Fritsche *et al.* 2008; Kanda *et al.* 2010; Friedrich *et al.* 2011). Our analysis of recombinant haplotypes in the currently largest available data set of AMD patients and controls has now refined the associated interval pointing to associated variants close to *ARMS2* but excluding variants near the *HTRA1* locus from disease association. In particular, the two synonymous variants in the first exon of *HTRA1* as well as the *HTRA1* promoter variant rs11200638 were excluded from AMD association, making it rather unlikely that *HTRA1* plays a significant causative role in AMD pathogenesis. This is also true for AMD-associated *HTRA1* variants rs1049331 and rs2293870, previously reported to strongly influence gene transcription (Yang *et al.* 2006) and more importantly its ability to bind insulin-like growth factor 1 (Jacobo *et al*. 2013) or to regulate TGFβ signaling (Friedrich *et al.* 2015).

Interestingly, the complex variant evs2663177 (del443ins54), which is located within the 3′-untranslated region (UTR) of *ARMS2* and which has been shown to influence stability of the *ARMS2* transcript (Fritsche *et al.* 2008), was also excluded by our analysis as being AMD associated. Therefore, a mechanism other than haploinsufficiency needs to be considered as disease related, involving the ARMS2 gene product. In line with this is an earlier notion emphasizing that variant ARMS2:rs2736911 resulting in a truncated ARMS2 protein (R38X) was never found to be associated with AMD (Friedrich *et al.* 2011), challenging the possibility of ARMS2 protein deficiency to have a role in AMD pathology.

In light of our findings, the most likely functional variant left is rs10490924 (p.A69S) in the *ARMS2* gene. Although this variant does not seem to strongly influence localization, stability, or expression of ARMS2 (Kanda *et al.* 2007; Wang *et al.* 2009; Kortvely *et al.* 2010), other data suggest that it could influence cell attachment *in vitro* (Cheng *et al*. 2013). Nevertheless, the true function and localization of ARMS2 still remains unclear, although our findings may put a new emphasis on clarifying the role of ARMS2 in the retina and, specifically, on testing functional consequences of the p.A69S polymorphism.

In conclusion, we demonstrate that genetic variants in or close to *ARMS2* but not *HTRA1* are responsible for disease susceptibility at the 10q26 locus. This finding will help to focus the functional analysis on *ARMS2* and its role in AMD pathogenesis.

## Supplementary Material

Supplemental material is available online at www.genetics.org/lookup/suppl/doi:10.1534/genetics.116.195966/-/DC1.
