# Supplementary material for: Recombinant Haplotypes Narrow the ARMS2/HTRA1 Association Signal for Age-Related Macular Degeneration
Source: Genetics. 2016 Nov 21;205(2):919–24. doi: 10.1534/genetics.116.195966 (PMC5289859; doi:10.1534/genetics.116.195966)
Supplement: Supplementary file 1 [file 919File1.docx]

**Table S1.** Haplotype association results accounting for phasing uncertainty

|  |  |  |  |  | **Frequency^b^ in** | |
| --- | --- | --- | --- | --- | --- | --- |
| **Haplotype #** | **Haplotype** | **Haplotype composition^a^** | **OR (95% CI)** | **P** | **Controls** | **Cases** |
| H0 | T-T-G-A-C-T-C-G-G-G-A-T-T-C-T-443bp-C-A-G-C-G-G-A-G-G | 0-0-0-0-0-0-0-0-0-0-0-0-0-0-0-0-0-0-0-0-0-0-0-0-0 | baseline | baseline | 0.771974 | 0.547276 |
| H1 | T-T-G-A-C-T-C-G-G-G-A-T-T-C-T-443bp-C-A-G-C-G-G-A-G-C | 0-0-0-0-0-0-0-0-0-0-0-0-0-0-0-0-0-0-0-0-0-0-0-0-1 | 0.81 (0.36;1.73) | 0.589245 | 0.000682 | 0.000437 |
| H2 | T-T-G-A-C-T-C-G-G-G-A-T-T-C-T-443bp-C-A-G-C-G-G-C-A-C | 0-0-0-0-0-0-0-0-0-0-0-0-0-0-0-0-0-0-0-0-0-0-1-1-1 | 1.63 (0.99;2.57) | 0.056148 | 0.001518 | 0.001651 |
| H3 | T-T-G-A-C-T-C-G-G-G-A-T-T-C-T-443bp-C-A-G-C-G-T-C-A-C | 0-0-0-0-0-0-0-0-0-0-0-0-0-0-0-0-0-0-0-0-0-1-1-1-1 | 0.89 (0.57;1.36) | 0.588704 | 0.002126 | 0.001425 |
| H4 | T-T-G-A-C-T-C-G-G-G-A-T-T-C-T-54bp-G-G-A-T-T-T-C-A-C | 0-0-0-0-0-0-0-0-0-0-0-0-0-0-0-1-1-1-1-1-1-1-1-1-1 | 0.82 (0.50;1.33) | 0.435261 | 0.001559 | 0.001118 |
| H5 | T-T-A-G-T-C-T-T-GGT-T-G-C-G-T-C-54bp-G-G-A-T-T-T-C-A-C | 0-0-1-1-1-1-1-1-1-1-1-1-1-1-1-1-1-1-1-1-1-1-1-1-1 | 4.47 (2.10;10.4) | 0.000205 | 0.000276 | 0.000875 |
| H6 | T-C-A-G-T-C-T-T-GGT-T-G-C-G-T-C-54bp-G-G-A-T-T-T-C-A-C | 0-1-1-1-1-1-1-1-1-1-1-1-1-1-1-1-1-1-1-1-1-1-1-1-1 | 2.76 (2.28;3.36) | 1.55E-18 | 0.005264 | 0.011234 |
| H7 | C-C-A-G-T-C-T-T-GGT-T-G-C-G-T-C-443bp-C-A-G-C-G-G-A-G-G | 1-1-1-1-1-1-1-1-1-1-1-1-1-1-1-0-0-0-0-0-0-0-0-0-0 | 3.52 (2.41;5.21) | 1.47E-10 | 0.001342 | 0.002814 |
| H8 | C-C-A-G-T-C-T-T-GGT-T-G-C-G-T-C-54bp-G-A-G-C-G-G-A-G-G | 1-1-1-1-1-1-1-1-1-1-1-1-1-1-1-1-1-0-0-0-0-0-0-0-0 | 2.70 (1.99;3.70) | 3.41E-10 | 0.002138 | 0.004075 |
| H9 | C-C-A-G-T-C-T-T-GGT-T-G-C-G-T-C-54bp-G-G-A-T-T-G-A-G-G | 1-1-1-1-1-1-1-1-1-1-1-1-1-1-1-1-1-1-1-1-1-0-0-0-0 | 2.78 (1.72;4.59) | 0.000043 | 0.001068 | 0.001958 |
| H10 | C-C-A-G-T-C-T-T-GGT-T-G-C-G-T-C-54bp-G-G-A-T-T-T-A-G-G | 1-1-1-1-1-1-1-1-1-1-1-1-1-1-1-1-1-1-1-1-1-1-0-0-0 | 3.15 (2.48;4.02) | 9.36E-21 | 0.003415 | 0.007881 |
| H11 | C-C-A-G-T-C-T-T-GGT-T-G-C-G-T-C-54bp-G-G-A-T-T-T-C-A-G | 1-1-1-1-1-1-1-1-1-1-1-1-1-1-1-1-1-1-1-1-1-1-1-1-0 | 3.71 (2.46;5.71) | 9.56E-10 | 0.001103 | 0.002741 |
| H12 | C-C-A-G-T-C-T-T-GGT-T-G-C-G-T-C-54bp-G-G-A-T-T-T-C-A-C | 1-1-1-1-1-1-1-1-1-1-1-1-1-1-1-1-1-1-1-1-1-1-1-1-1 | 2.86 (2.75;2.97) | 3.33E-640 | 0.188783 | 0.392989 |

^a^ 0 indicates non-risk allele, 1 indicates risk increasing allele (see Table 1)

^b^ average haplotype frequency in cases or controls over 100 phasing/imputation runs
