# Supplementary material for: Recombinant Haplotypes Narrow the ARMS2/HTRA1 Association Signal for Age-Related Macular Degeneration
Source: Genetics. 2016 Nov 21;205(2):919–24. doi: 10.1534/genetics.116.195966 (PMC5289859; doi:10.1534/genetics.116.195966)
Supplement: Supplementary file 2 [file 919File2.pdf]

Recombinant haplotypes narrow the ARMS2/HTRA1 association signal for age-related macular degeneration

Felix Grassmann (email: felix.grassmann@klinik.uni-regensburg.de)[1], Iris M. Heid (email: iris.heid@klinik.uni-regensburg.de)[2], International AMD Genomics Consortium (IAMDGC)[§], and Bernhard H.F. Weber (email: bweb@klinik.uni-regensburg.de)[1,‡]

[1]Institute for Human Genetics, University of Regensburg, Regensburg, Germany
[2]Department of Genetic Epidemiology, University of Regensburg, Regensburg, Germany

[§] Members of the IAMDGC:

Fritsche LG, Igl W, Bailey JN, Grassmann F, Sengupta S, Bragg-Gresham JL, Burdon KP, Hebbring SJ, Wen C, Gorski M, Kim IK, Cho D, Zack D, Souied E, Scholl HP, Bala E, Lee KE, Hunter DJ, Sardell RJ, Mitchell P, Merriam JE, Cipriani V, Hoffman JD, Schick T, Lechanteur YT, Guymer RH, Johnson MP, Jiang Y, Stanton CM, Buitendijk GH, Zhan X, Kwong AM, Boleda A, Brooks M, Gieser L, Ratnapriya R, Branham KE, Foerster JR, Heckenlively JR, Othman MI, Vote BJ, Liang HH, Souzeau E, McAllister IL, Isaacs T, Hall J, Lake S, Mackey DA, Constable IJ, Craig JE, Kitchner TE, Yang Z, Su Z, Luo H, Chen D, Ouyang H, Flagg K, Lin D, Mao G, Ferreyra H, Stark K, von Strachwitz CN, Wolf A, Brandl C, Rudolph G, Olden M, Morrison MA, Morgan DJ, Schu M, Ahn J, Silvestri G, Tsironi EE, Park KH, Farrer LA, Orlin A, Brucker A, Li M, Curcio CA, Mohand-Saïd S, Sahel JA, Audo I, Benchaboune M, Cree AJ, Rennie CA, Goverdhan SV, Grunin M, Hagbi-Levi S, Campochiaro P, Katsanis N, Holz FG, Blond F, Blanché H, Deleuze JF, Igo RP Jr, Truitt B, Peachey NS, Meuer SM, Myers CE, Moore

EL, Klein R, Hauser MA, Postel EA, Courtenay MD, Schwartz SG, Kovach JL, Scott WK, Liew G, Tan AG, Gopinath B, Merriam JC, Smith RT, Khan JC, Shahid H, Moore AT, McGrath JA, Laux R, Brantley MA Jr, Agarwal A, Ersoy L, Caramoy A, Langmann T, Saksens NT, de Jong EK, Hoyng CB, Cain MS, Richardson AJ, Martin TM, Blangero J, Weeks DE, Dhillon B, van Duijn CM, Doheny KF, Romm J, Klaver CC, Hayward C, Gorin MB, Klein ML, Baird PN, den Hollander AI, Fauser S, Yates JR, Allikmets R, Wang JJ, Schaumberg DA, Klein BE, Hagstrom SA, Chowers I, Lotery AJ, Léveillard T, Zhang K, Brilliant MH, Hewitt AW, Swaroop A, Chew EY, Pericak-Vance MA, DeAngelis M, Stambolian D, Haines JL, Iyengar SK, Weber BH, Abecasis GR, Heid IM.

‡ Corresponding author:

Bernhard H. F. Weber, Ph.D.

Institute of Human Genetics, University of Regensburg

Franz-Josef-Strauss-Allee 11

D-93053 Regensburg, Germany

Phone: (+49) 941 944 5400; Fax: (+49) 941 944 5402;

E-mail: bweb@klinik.uni-regensburg.de

Key words: Age-Related Macular Degeneration; genetic association studies; linkage disequilibrium; haplotypes; ARMS2/HTRA1 gene locus

Article Summary: Age-related macular degeneration (AMD) is the leading cause of blindness in ageing societies triggered by both environmental and genetic factors. The strongest genetic signal for AMD was found over a chromosomal region in 10q26

harboring two genes, ARMS2 and HTRA1, although with little knowledge as to which gene is functionally relevant to AMD pathology. In this study, we analyzed rare recombinant haplotypes in 16,144 AMD cases and 17,832 controls and identified variants in ARMS2 but not HTRA1 to exclusively carry the AMD risk. This now allows prioritization of the gene of interest for subsequent functional studies.

Abstract

Age-related macular degeneration (AMD) is the leading cause of blindness in ageing societies and is caused by both environmental and genetic factors. The strongest genetic signal for AMD with odds ratios of up to 2.8 per adverse allele was found previously to center over two genes, *ARMS2* and *HTRA1* on 10q26, although with little knowledge as to the true functionally relevant gene or genetic variation. Due to extensive linkage disequilibrium (LD) at this locus, it was long assumed that the broad association signal cannot be dissected by statistical means. In this report, we have now separated the 10q26 region by analyzing imputed haplotypes with the help of a large case/control association data set. Rare recombinant haplotypes identified in 16,144 cases and 17,832 controls from the International AMD Genomics Consortium (IAMDGC), revealed genetic variants in *ARMS2* but not *HTRA1* to exclusively carry the AMD disease load with p values between 1.0×10^{-773} and 6.7×10^{-5}. This study is a proof-of-concept and universally applicable to refine extended association signals providing the means to prioritize genes of interest for subsequent biological studies.

Introduction

Age-related macular degeneration (AMD) is a prevalent cause of severe vision loss in ageing societies with a strong component of genetic predisposition. Genome-wide association studies and large scale re-sequencing initiatives have identified a large number of single nucleotide variants (SNVs) enriched in complement and complement related genes that confer a strong risk for AMD. Recently, the International AMD Genomics Consortium (IAMDGC) (Fritsche *et al.* 2016) reported 34 independent AMD risk loci, together explaining approximately 50% of disease heritabilty.

Among the strongest loci associated with AMD are the complement factor H (CFH) locus on chromosome 1q32 and a region on chromosome 10q26 harboring two genes, namely age-related maculopathy susceptibility 2 (*ARMS2*) and HtrA serine peptidase 1 (*HTRA1*) (Jakobsdottir *et al.* 2005; Rivera *et al.* 2005). While the causative genes at the CFH site appear undisputed, the *ARMS2/HTRA1* region proved notoriously difficult to dissect by statistical means as the chromosomal region displays correlated variants in high linkage disequilibrium (LD) (Dewan *et al.* 2006; Fritsche *et al.* 2008; Yang *et al.* 2010; Kanda *et al.* 2010; Friedrich *et al.* 2011, 2015). In addition, as both *ARMS2* and *HTRA1* harbor functional variants which can be related to relevant disease processes, so far, it is unclear to which gene the observed disease association can functionally be attributed (Dewan *et al.* 2006; Fritsche *et al.* 2008; Cheng *et al.* 2013; Friedrich *et al.* 2015).

Despite the strong LD in the *ARMS2/HTRA1* interval, the region still exhibits some level of recombination resulting in rare recombinant haplotypes. Similar to gene mapping in monogenic diseases, recombinant haplotypes can be helpful in dissecting a disease-associated genomic region. To this end, we used the currently largest dataset on AMD genetics including over 33,000 genotyped individuals (Fritsche *et al.* 2016) and

analyzed the rare, but informative recombinant haplotypes on 10q26 to define a minimal set of variants associated with AMD.

Material and Methods

Ethics Statement

The study followed the tenets of the Declaration of Helsinki and was approved by the local Ethics Review Board at participating sites, as previously described (Fritsche *et al.* 2016). Informed written consent was obtained from each patient after explanation of the nature and possible consequences of the study.

Study data and data availability

The genotypes of this study are available from the database of Genotypes and Phenotypes (dbGAP) under accession phs001039.v1.p1 while GWAS summary statistics are available at http://amdgenetics.org/. Our data consist of 16,144 late stage AMD cases and 17,832 AMD-free controls from European ancestry, all unrelated, as published previously (Fritsche *et al.* 2016). Inclusion and exclusion criteria as well as detailed information on ophthalmological grading, quality control of genetic data and imputation are given elsewhere (Fritsche *et al.* 2016). Genotyping and imputation using the 1000 Genomes reference panel (Abecasis *et al.* 2012) was performed as described previously (Fritsche *et al.* 2016). Briefly, we extracted the genotypes of all variants in the *ARMS2/HTRA* locus, defined as the region of 1.25 million basepairs around the previously described lead variant rs3750846. The extracted genotypes were phased with ShapeIt2 following standard settings (O'Connell *et al.* 2014). The resulting haplotypes were then used for imputation with IMPUTE2 (Rahpeymai *et al.* 2006), utilizing the 1000 Genomes reference panel. Upon imputation, IMPUTE2 generates best-guess imputed haplotypes, which can be outputted using the *-haps* command. We used these haplotypes for further haplotype analyses. In addition, the imputed

genotypes were coded as dosage data ranging from 0 to 2 for single variant association testing as well as mediation analyses. In total, 3.446 variants were either genotyped or could be imputed reliably in the *ARMS2/HTRA1* region.

Several DNA specimens had been genotyped after whole genome amplification (WGA). To account for possible confounding effects of WGA, a categorical variable "WGA" was computed indicating the presence or absence of whole genome amplification in all samples. Additionally, the first two principle components (PC1 and PC2) were computed from all genotyped variants as described previously (Fritsche *et al.* 2016). All logistic regression models were adjusted for WGA and PC1 and PC2 to account for different DNA source and potential population stratification.

Searching for additional signals at the ARMS2/HTRA1 locus

All statistical procedures were carried out as implemented in R. Step-wise conditional logistic regression analysis on the imputed dosages was used previously and failed to detect additional independent signals at the *ARMS2/HTRA1* locus (Fritsche *et al.* 2016). We also aimed to exclude the possibility that the main signal represented by rs3750846 was explained by two causal variants where the risk carrying alleles are inherited together with one of the rs3750846 alleles. In such a scenario the two causal variants would represent two or more haplotypes, which would be tagged by one of the alleles of rs3750846 (Grassmann *et al.* 2012; Fritsche *et al.* 2016). We used mediation analysis to test each pair of variants in the region for jointly explaining the main signal: the beta-estimate of the main variant (the rs3750846) in a logistic regression model without adjusting for the pair is compared to the respective beta-estimate with adjusting for the pair of variants (Imai *et al.* 2010), using the *mediate* function from the *mediation* package in R (Tingley *et al.* 2014).

For all analyses, we utilized the genotypes determined experimentally or the dosages in the case of imputed variants. In case of a significant mediation, the effect size of the main variant would be expected to drop strongly and significantly. To obtain reliable non-parametric P-value estimates, we calculated one thousand bootstrap replicates and adjusted the resulting P-values according to the False-Discovery Rate. To reduce the complexity of the mediation analysis, we first extracted variants in linkage with rs3750846 by computing D' using the haplotypes obtained from the imputed genotypes and extracted all variants with D' > 0.8. This should effectively capture the relevant common haplotypes tagged by rs3750846.

Best guess haplotype association analyses

To further investigate the AMD associated haplotypes at the *ARMS2/HTRA1* locus, we extracted the relevant variants. To capture the most likely causal variant (van de Bunt *et al.* 2015) , we included variants that were correlated to rs3750846 ($R^2 > 0.8$) as well as all variants that were included in the 99% credible set of associated variants (Maller *et al.* 2012; Fritsche *et al.* 2016).The 99% credible set of associated variants was computed from the Z-scores of all variants at this locus (Kichaev *et al.* 2014), effectively capturing the variants with the strongest evidence for association. The best-guess haplotypes defined by these variants (i.e. haplotypes that carry alleles of these variants) were investigated for their association with AMD using multivariable logistic regression models including all haplotypes with reasonable counts (≥ 34) in the study sample except the non-risk allele carrying haplotype (H0), which served as reference. We assessed the association of the haplotypes using a logistic regression model including all haplotypes. Haplotype H0 which carried exclusively non-risk increasing alleles served as baseline. We excluded variants to be disease associated if either the non-risk increasing alleles (protective alleles) of the variant were present on haplotypes that

increase the risk for AMD or if the risk increasing alleles were present on protective haplotypes or on haplotypes not associated with AMD.

Accounting for phase uncertainty

To determine haplotype phase, the calculation of several plausible haplotypes for each individual is required. This process is repeated many times and eventually returns the best guess haplotypes for each individual. It thus is possible that the estimated best guess haplotypes are only slightly more likely than other haplotypes and that random effects may come into play. In order to account for this uncertainty, we repeated the phasing of the haplotypes and the subsequent imputation of the *ARMS2/HTRA1* locus one hundred times and mapped the occurance of the resulting haplotypes to the best guess haplotypes. As such, each individual is characterized by the frequency or dosage of the 13 haplotypes (ranging from 0 to 2). The haplotype dosages were then analysed for their association with AMD using the function haplo.glm from the package haplo.stats in R (Lake *et al.* 2003).

Results

The present study included 16,144 late stage AMD cases and 17,832 AMD-free controls with both groups of European ancestry (Fritsche *et al.* 2016). Variant and sample quality control as well as imputation based on the 1000G reference panel (Abecasis *et al.* 2012) was reported previously (Fritsche *et al.* 2016). Variant rs3750846 residing within intron 1 of *ARMS2* exhibited the strongest association with AMD on 10q26 and is referred to as lead variant (Fritsche *et al.* 2016). We defined the *ARMS2/HTRA1* locus as a region within 1.25 million basepairs around rs3750846 to assure the capture of all potentially correlated and associated variants.

Initially, we explored the number of causal variants explaining the association signal at *ARMS2/HTRA1*. When applying step-wise logistic regression as described previously (Fritsche *et al.* 2016), there was only one but no independent second signal at this locus. There was also no pair of variants that jointly explained the main signal when applying mediator analyses. Together, these findings suggested that the association at this locus was confered to by a single haplotype tagged by the lead variant rs3750846.

We then narrowed the region of interest by haplotype analysis focussing on highly correlated variants. The best guess haplotypes for the locus were derived and their complexity was reduced by focussing on 25 variants that were (i) highly correlated with rs3750846 ($R^2 > 0.8$) or (ii) were in the 99% credible set of associated variants (Maller *et al.* 2012; van de Bunt *et al.* 2015; Fritsche *et al.* 2016) (**Table 1**). This resulted in 13 haplotypes that were counted at least 34 times in the study sample and thus resulted in a haplotype frequency ≥ 0.05% (**Figure 1**). Twelve haplotypes were included in a haplotype association analysis by logistic regression modeling with the common non-risk allele representing haplotype H0 as reference (**Figure 1**). As expected, the common haplotype carrying all risk increasing alleles (H12) was strongly associated with AMD ($P < 10^{-50}$).

Two additional findings were of particular interest when focusing on the rare recombinant haplotypes: (1) haplotypes H1 to H4 with risk increasing alleles exclusively at the variants downstream of rs3750846 were not associated with AMD ($P > 0.05$) (**Figure 1**), while (2) haplotypes H7 to H11 with the non-risk alleles at the variants downstream of rs3750846 were highly significantly associated with AMD (P from $<10^{-50}$ to 6.68×10^{-5}). In addition, the first two variants upstream of ARMS2 (rs61871744 and rs11200630) were also not associated with AMD, since haplotypes without the risk increasing alleles at the two variants were associated with increased disease risk.

Together, these findings reveal that variants downstream of the lead variant rs3750846 do not contribute to AMD risk and that the most likely candidates driving the AMD association are 13 variants in and immediatley upstream of the *ARMS2* gene. A further narrowing of the refined AMD-associated interval would require an even larger data set due to the low rate of recombination between the local variants at and around ARMS2.

Our estimation of haplotypes and subsequent imputations relied on the calculation of best guess haplotypes derived from several likely haplotypes calculated over several (internal) iterations. However, some of those recombinant haplotypes could in fact be better represented by less likely haplotypes, which were not chosen initially due to random effects. We therefore repeated the phasing and impuation one hundred times to account for phasing uncertainty. The resulting haplotype occurances were mapped to the 13 best guess haplotypes and analysed using logistic regression. The results of this analysis was similar to the results obtained from the best guess haplotypes (Supplemental Material, **Table S1**), indicating that the reconstructed best guess haplotypes are robust and likely represent the true haplotype structure in our cohort.

Discussion

Since its initial reporting, the *ARMS2/HTRA1* region has been a point of controversy as to which gene is causally linked to AMD pathogenesis (Yang *et al.* 2006; Fritsche *et al.* 2008; Kanda *et al.* 2010; Friedrich *et al.* 2011). Our analysis of recombinant haplotypes in the currently largest available dataset of AMD patients and controls has now refined the associated interval pointing to associated variants close to *ARMS2* but excluding variants near the *HTRA1* locus from disease association. In particular, the two synonymous variants in the first exon of *HTRA1* as well as the *HTRA1* promoter variant rs11200638 were excluded from AMD association making it rather unlikely that *HTRA1*

plays a significant causative role in AMD pathogenesis. This is also true for AMD-associated *HTRA1* variants rs1049331 and rs2293870, previously reported to strongly influence gene transcription (Yang *et al.* 2006) and more importantly its ability to bind insulin-like growth factor 1 or to regulate TGFß signalling (Friedrich *et al.* 2015).

Interestingly, the complex variant evs2663177 (del443ins54), which is located within the 3'-untranslated region of *ARMS2* and which has been shown to influence stability of the *ARMS2* transcript (Fritsche *et al.* 2008), was also excluded by our analysis as being AMD-associated. Therefore, a mechanism other than haploinsufficiency needs to be considered as disease related involving the ARMS2 gene product. In line with this is an earlier notion emphasizing that variant ARMS2:rs2736911 resulting in a truncated ARMS2 protein (R38X) was never found to be associated with AMD (Friedrich *et al.* 2011) challenging the possibility of ARMS2 protein deficiency to have a role in AMD pathology.

In light of our findings, the most likely functional variant left is rs10490924 (p.A69S) in the *ARMS2* gene. Although this variant does not seem to strongly influence localization, stability or expression of ARMS2 (Kanda *et al.* 2007; Wang *et al.* 2009; Kortvely *et al.* 2010), other data suggest that it could influence cell attachment *in vitro* [10.1371/journal.pone.0053665]. Nevertheless, the true function and localisation of ARMS2 still remains unclear, although our findings may put a new emphasis on clarifying the role of ARMS2 in the retina and, specifically, on testing functional consequences of the p.A69S polymorphism.

In concluding, we demonstrate that genetic variants in or close to *ARMS2* but not *HTRA1* are responsible for disease susceptibility at the 10q26 locus. This finding will help to focus the functional analysis on *ARMS2* and its role in AMD pathogenesis.

Literature Cited

Abecasis, G. R., A. Auton, L. D. Brooks, M. A. DePristo, R. M. Durbin, *et al.*, 2012 An integrated map of genetic variation from 1,092 human genomes. Nature **491**: 56–65.

Bunt, M. van de, A. Cortes, M. A. Brown, A. P. Morris, and M. I. McCarthy, 2015 Evaluating the Performance of Fine-Mapping Strategies at Common Variant GWAS Loci (Y-Y Teo, Ed.). PLOS Genet. **11**: e1005535.

Cheng, Y., L. Huang, X. Li, P. Zhou, W. Zeng, *et al.*, 2013 Genetic and Functional Dissection of ARMS2 in Age-Related Macular Degeneration and Polypoidal Choroidal Vasculopathy (H-C Lee, Ed.). PLoS One **8**: e53665.

Dewan, A., M. Liu, S. Hartman, S. S.-M. Zhang, D. T. L. Liu, *et al.*, 2006 HTRA1 promoter polymorphism in wet age-related macular degeneration. Science **314**: 989–92.

Friedrich, U., C. a. Myers, L. G. Fritsche, A. Milenkovich, A. Wolf, *et al.*, 2011 Risk and non risk associated variants at the 10q26 AMD locus influence ARMS2 mRNA expression but exclude pathogenic effects due to protein deficiency. Hum. Mol. Genet. **20**: 1387–1399.

Friedrich, U., S. Datta, T. Schubert, K. Plössl, M. Schneider, *et al.*, 2015 Synonymous variants in HTRA1 implicated in AMD susceptibility impair its capacity to regulate TGF-β signaling. Hum. Mol. Genet. **24**: 6361–73.

Fritsche, L. G., T. Loenhardt, A. Janssen, S. a Fisher, A. Rivera, *et al.*, 2008 Age-related macular degeneration is associated with an unstable ARMS2 (LOC387715) mRNA. Nat. Genet. **40**: 892–6.

Fritsche, L. G., W. Igl, J. N. C. Bailey, F. Grassmann, S. Sengupta, *et al.*, 2016 A large

genome-wide association study of age-related macular degeneration highlights contributions of rare and common variants. Nat. Genet. **48**: 134–43.

Grassmann, F., L. G. Fritsche, C. N. Keilhauer, I. M. Heid, and B. H. F. Weber, 2012 Modelling the genetic risk in age-related macular degeneration. PLoS One **7**: e37979.

Imai, K., L. Keele, and D. Tingley, 2010 A general approach to causal mediation analysis. Psychol. Methods **15**: 309–334.

Jakobsdottir, J., Y. P. Conley, D. E. Weeks, T. S. Mah, R. E. Ferrell, *et al.*, 2005 Susceptibility genes for age-related maculopathy on chromosome 10q26. Am. J. Hum. Genet. **77**: 389–407.

Kanda, A., W. Chen, M. Othman, K. E. H. Branham, M. Brooks, *et al.*, 2007 A variant of mitochondrial protein LOC387715/ARMS2, not HTRA1, is strongly associated with age-related macular degeneration. Proc. Natl. Acad. Sci. U. S. A. **104**: 16227–32.

Kanda, A., D. Stambolian, W. Chen, C. A. Curcio, G. R. Abecasis, *et al.*, 2010 Age-related macular degeneration-associated variants at chromosome 10q26 do not significantly alter ARMS2 and HTRA1 transcript levels in the human retina. Mol. Vis. **16**: 1317–23.

Kichaev, G., W.-Y. Yang, S. Lindstrom, F. Hormozdiari, E. Eskin, *et al.*, 2014 Integrating functional data to prioritize causal variants in statistical fine-mapping studies. PLoS Genet. **10**: e1004722.

Kortvely, E., S. M. Hauck, G. Duetsch, C. J. Gloeckner, E. Kremmer, *et al.*, 2010 ARMS2 Is a Constituent of the Extracellular Matrix Providing a Link between Familial and Sporadic Age-Related Macular Degenerations. Investig. Opthalmology Vis. Sci. **51**: 79.

Lake, S. L., H. Lyon, K. Tantisira, E. K. Silverman, S. T. Weiss, *et al.*, 2003 Estimation and Tests of Haplotype-Environment Interaction when Linkage Phase Is Ambiguous. Hum. Hered. **55**: 56–65.

Maller, J. B., G. McVean, J. Byrnes, D. Vukcevic, K. Palin, *et al.*, 2012 Bayesian refinement of association signals for 14 loci in 3 common diseases. Nat. Genet. **44**: 1294–301.

O'Connell, J., D. Gurdasani, O. Delaneau, N. Pirastu, S. Ulivi, *et al.*, 2014 A general approach for haplotype phasing across the full spectrum of relatedness. PLoS Genet. **10**: e1004234.

Rahpeymai, Y., M. A. Hietala, U. Wilhelmsson, A. Fotheringham, I. Davies, *et al.*, 2006 Complement: a novel factor in basal and ischemia-induced neurogenesis. EMBO J. **25**: 1364–74.

Rivera, A., S. A. Fisher, L. G. Fritsche, C. N. Keilhauer, P. Lichtner, *et al.*, 2005 Hypothetical LOC387715 is a second major susceptibility gene for age-related macular degeneration, contributing independently of complement factor H to disease risk. Hum. Mol. Genet. **14**: 3227–36.

Tingley, D., T. Yamamoto, K. Hirose, L. Keele, and K. Imai, 2014 mediation : R Package for Causal Mediation Analysis. J. Stat. Softw. **59**.

Wang, G., K. L. Spencer, B. L. Court, L. M. Olson, W. K. Scott, *et al.*, 2009 Localization of age-related macular degeneration-associated ARMS2 in cytosol, not mitochondria. Invest. Ophthalmol. Vis. Sci. **50**: 3084–90.

Yang, Z., N. J. Camp, H. Sun, Z. Tong, D. Gibbs, *et al.*, 2006 A variant of the HTRA1 gene increases susceptibility to age-related macular degeneration. Science **314**: 992–3.

Yang, Z., Z. Tong, Y. Chen, J. Zeng, F. Lu, *et al.*, 2010 Genetic and Functional Dissection of HTRA1 and LOC387715 in Age-Related Macular Degeneration. PLoS Genet. **6**: 1–9.

Figure Legends:

Figure 1. Delineating associated haplotypes at the *ARMS2/HTRA1* locus. Shown are 13 haplotypes defined by 25 variants that were either correlated with the lead variant rs3750846 (R^2> 0.8) or in the 99% credible set of associated variants (Fritsche *et al.* 2016) (see also **Table 1**). P-values are given based on a logistic regression model including H1-H12 as covariates and H0 carrying exclusively non-risk alleles as reference. AMD risk increasing alleles are colored in red and the protective alleles in blue. In case a haplotype is carrying the risk increasing allele of a variant but is not singificantly associated with AMD, this variant is regarded not to be associated with disease risk. Similarly, if a haplotype carrying the protective variant allele is increasing the risk for the disease, we concluded that this variant is not associated with AMD risk. The minimal set of risk-associated variants (grey box) includes 13 variants not excluded by the haplotype analysis and located exclusively at the immediate *ARMS2* locus. Thin dark blue boxes represent the UTR, thick dark blue boxes represent the coding region of *ARMS2* as well as the first exon of *HTRA1*. Gene sizes and relative positions are not to scale. Intronic sequences are given by dark blue arrows indicating the direction of transcription. Each square as part of a haplotype represents a single variant, ordered by chromosomal position from centromere (left) to telomere (right). Also shown is the number of observed best-guess haplotypes (N), the odds ratio (OR) and 95% confidence intervals (95% CI) as well as the frequency of the best-guess haplotypes H0-H12 in cases and controls.

Table 1. Candidate variants at the *ARMS2/HTRA1* locus on 10q26 correlated with rs3750846 (R^2>0.8) or part of the 99% credible set of associated variants.

Position on	rs ID	Non-risk	Risk	Location/Consequence	-log10	PPA [a]	R^2 [b]	D' [b]	OR (95% CI) [c]	excluded
124203787	rs61871744	T	C	intergenic	741.2	1.83E-42	0.952	0.995	2.787 (2.693;2.885)	yes
124209684	rs11200630	T	C	intergenic	774.5	3.56E-09	0.989	0.999	2.798 (2.706;2.894)	yes
124210369	rs61871745	G	A	intergenic	780.4	0.003	0.996	0.999	2.804 (2.711;2.900)	no
124211536	rs11200632	A	G	intergenic	780.2	0.002	0.998	0.999	2.796 (2.703;2.891)	no
124211596	rs11200633	C	T	intergenic	780.3	0.002	0.998	0.999	2.795 (2.703;2.890)	no
124212913	rs61871746	T	C	intergenic	781.3	0.024	0.998	1.000	2.794 (2.702;2.889)	no
124213046	rs61871747	C	T	intergenic	781.5	0.033	0.998	1.000	2.793 (2.701;2.888)	no
124214448[d]	rs10490924	G	T	ARMS2: p.A69S	781.8	0.076	0.998	1.000	2.786 (2.694;2.881)	no
124214600	10:124214600	G	GGT	ARMS2: intronic	781.9	0.091	0.998	1.000	2.787 (2.695;2.881)	no
124214976	rs36212731	G	T	ARMS2: intronic	781.9	0.094	0.999	1.000	2.787 (2.695;2.881)	no
124215198[d]	rs36212732	A	G	ARMS2: intronic	781.9	0.085	0.999	1.000	2.786 (2.695;2.881)	no
124215211	rs36212733	T	C	ARMS2: intronic	782.0	0.110	0.999	1.000	2.787 (2.695;2.881)	no
124215315	rs3750848	T	G	ARMS2: intronic	782.1	0.139	0.999	1.000	2.787 (2.695;2.882)	no
124215421[d]	rs3750847	C	T	ARMS2: intronic	782.2	0.168	0.999	1.000	2.787 (2.695;2.882)	no
124215565	rs3750846	T	C	ARMS2: intronic	782.2	0.173	1.000	1.000	2.787 (2.695;2.882)	no
124216820	esv2663177	443bp	54bp	ARMS2: UTR del442ins54	770.0	1.02E-13	0.981	0.991	2.762 (2.671;2.856)	yes
124219275[d]	rs3793917	C	G	intergenic	768.0	1.10E-15	0.979	0.990	2.756 (2.665;2.849)	yes
124220061	rs3763764	A	G	intergenic	759.6	4.05E-24	0.965	0.989	2.746 (2.656;2.839)	yes
124220544[d]	rs11200638	G	A	HTRA1: Promoter	755.9	9.29E-28	0.963	0.988	2.737 (2.647;2.830)	yes
124221270	rs1049331	C	T	HTRA1: p.A34A	755.6	5.06E-28	0.961	0.987	2.744 (2.654;2.838)	yes
124221276	rs2293870	G	T	HTRA1: p.G36G	755.5	3.44E-28	0.961	0.987	2.744 (2.654;2.838)	yes
124226630	rs2284665	G	T	HTRA1: intronic	742.4	3.10E-41	0.933	0.974	2.724 (2.634;2.817)	yes
124230024	rs58077526	A	C	HTRA1: intronic	716.7	5.71E-67	0.896	0.958	2.672 (2.584;2.763)	yes
124231464	rs932275	G	A	HTRA1: intronic	716.7	5.23E-67	0.899	0.962	2.678 (2.590;2.770)	yes
124234037	rs2142308	G	C	HTRA1: intronic	711.1	1.45E-72	0.888	0.956	2.666 (2.578;2.756)	yes

[a] Posterior probability of association
[b] Linkage to the top variant rs3750846
[c] Odds ratio and 95% confidence intervals of risk increasing allele
[d] directly genotyped variant

Table S1. Haplotype association results accounting for phasing uncertainty

					Frequency[b] in	
**Haplotype #**	**Haplotype**	**Haplotype composition[a]**	**OR (95% CI)**	**P**	**Controls**	**Cases**
H0	T-T-G-A-C-T-C-G-G-G-A-T-T-C-T-443bp-C-A-G-C-G-G-A-G-G	0-0-0-0-0-0-0-0-0-0-0-0-0-0-0-0-0-0-0-0-0-0-0-0-0	baseline	baseline	0.771974	0.547276
H1	T-T-G-A-C-T-C-G-G-G-A-T-T-C-T-443bp-C-A-G-C-G-G-A-G-C	0-0-0-0-0-0-0-0-0-0-0-0-0-0-0-0-0-0-0-0-0-0-0-0-1	0.81 (0.36;1.73)	0.589245	0.000682	0.000437
H2	T-T-G-A-C-T-C-G-G-G-A-T-T-C-T-443bp-C-A-G-C-G-G-C-A-C	0-0-0-0-0-0-0-0-0-0-0-0-0-0-0-0-0-0-0-0-0-0-1-1-1	1.63 (0.99;2.57)	0.056148	0.001518	0.001651
H3	T-T-G-A-C-T-C-G-G-G-A-T-T-C-T-443bp-C-A-G-C-G-T-C-A-C	0-0-0-0-0-0-0-0-0-0-0-0-0-0-0-0-0-0-0-0-0-1-1-1-1	0.89 (0.57;1.36)	0.588704	0.002126	0.001425
H4	T-T-G-A-C-T-C-G-G-G-A-T-T-C-T-54bp-G-G-A-T-T-T-C-A-C	0-0-0-0-0-0-0-0-0-0-0-0-0-0-0-1-1-1-1-1-1-1-1-1-1	0.82 (0.50;1.33)	0.435261	0.001559	0.001118
H5	T-T-A-G-T-C-T-T-GGT-T-G-C-G-T-C-54bp-G-G-A-T-T-T-C-A-C	0-0-1-1-1-1-1-1-1-1-1-1-1-1-1-1-1-1-1-1-1-1-1-1-1	4.47 (2.10;10.4)	0.000205	0.000276	0.000875
H6	T-C-A-G-T-C-T-T-GGT-T-G-C-G-T-C-54bp-G-G-A-T-T-T-C-A-C	0-1-1-1-1-1-1-1-1-1-1-1-1-1-1-1-1-1-1-1-1-1-1-1-1	2.76 (2.28;3.36)	1.55E-18	0.005264	0.011234
H7	C-C-A-G-T-C-T-T-GGT-T-G-C-G-T-C-443bp-C-A-G-C-G-G-A-G-G	1-1-1-1-1-1-1-1-1-1-1-1-1-1-1-0-0-0-0-0-0-0-0-0-0	3.52 (2.41;5.21)	1.47E-10	0.001342	0.002814
H8	C-C-A-G-T-C-T-T-GGT-T-G-C-G-T-C-54bp-G-A-G-C-G-G-A-G-G	1-1-1-1-1-1-1-1-1-1-1-1-1-1-1-1-0-0-0-0-0-0-0-0-0	2.70 (1.99;3.70)	3.41E-10	0.002138	0.004075
H9	C-C-A-G-T-C-T-T-GGT-T-G-C-G-T-C-54bp-G-G-A-T-T-G-A-G-G	1-1-1-1-1-1-1-1-1-1-1-1-1-1-1-1-1-1-1-1-1-0-0-0-0	2.78 (1.72;4.59)	0.000043	0.001068	0.001958
H10	C-C-A-G-T-C-T-T-GGT-T-G-C-G-T-C-54bp-G-G-A-T-T-T-A-G-G	1-1-1-1-1-1-1-1-1-1-1-1-1-1-1-1-1-1-1-1-1-1-0-0-0	3.15 (2.48;4.02)	9.36E-21	0.003415	0.007881
H11	C-C-A-G-T-C-T-T-GGT-T-G-C-G-T-C-54bp-G-G-A-T-T-T-C-A-G	1-1-1-1-1-1-1-1-1-1-1-1-1-1-1-1-1-1-1-1-1-1-1-1-0	3.71 (2.46;5.71)	9.56E-10	0.001103	0.002741
H12	C-C-A-G-T-C-T-T-GGT-T-G-C-G-T-C-54bp-G-G-A-T-T-T-C-A-C	1-1-1-1-1-1-1-1-1-1-1-1-1-1-1-1-1-1-1-1-1-1-1-1-1	2.86 (2.75;2.97)	3.33E-640	0.188783	0.392989

[a] 0 indicates non-risk allele, 1 indicates risk increasing allele (see Table 1)

[b] average haplotype frequency in cases or controls over 100 phasing/imputation runs

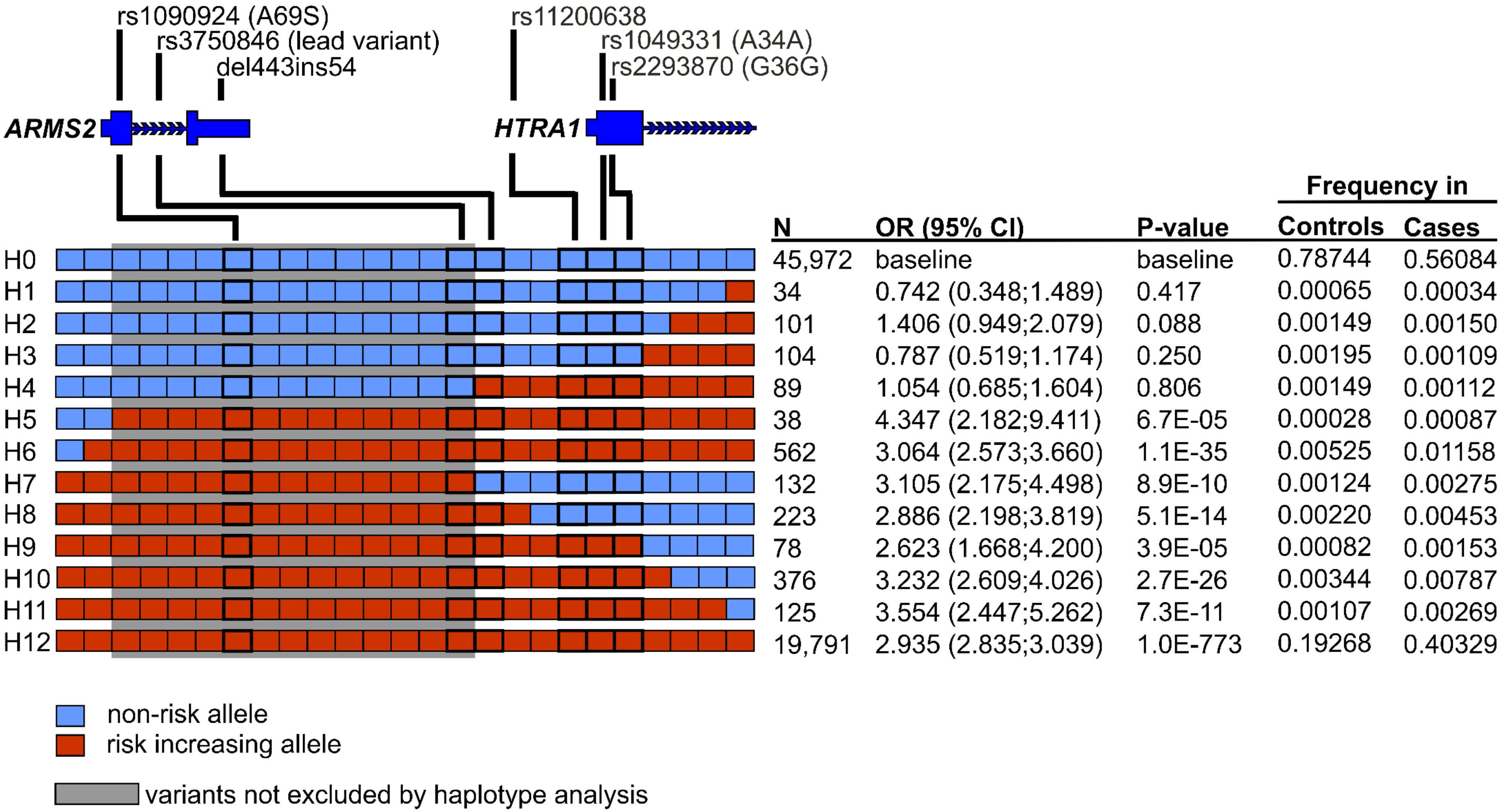

	N	OR (95% CI)	P-value	Frequency in Controls	Frequency in Cases
H0	45,972	baseline	baseline	0.78744	0.56084
H1	34	0.742 (0.348;1.489)	0.417	0.00065	0.00034
H2	101	1.406 (0.949;2.079)	0.088	0.00149	0.00150
H3	104	0.787 (0.519;1.174)	0.250	0.00195	0.00109
H4	89	1.054 (0.685;1.604)	0.806	0.00149	0.00112
H5	38	4.347 (2.182;9.411)	6.7E-05	0.00028	0.00087
H6	562	3.064 (2.573;3.660)	1.1E-35	0.00525	0.01158
H7	132	3.105 (2.175;4.498)	8.9E-10	0.00124	0.00275
H8	223	2.886 (2.198;3.819)	5.1E-14	0.00220	0.00453
H9	78	2.623 (1.668;4.200)	3.9E-05	0.00082	0.00153
H10	376	3.232 (2.609;4.026)	2.7E-26	0.00344	0.00787
H11	125	3.554 (2.447;5.262)	7.3E-11	0.00107	0.00269
H12	19,791	2.935 (2.835;3.039)	1.0E-773	0.19268	0.40329

Figure 1
